# The landscape of biofilm models for phage therapy: mimicking biofilms in diabetic foot ulcers using 3D models

**DOI:** 10.3389/fmicb.2025.1553979

**Published:** 2025-02-24

**Authors:** Mark Grevsen Martinet, Marvin Thomas, Jörg Bojunga, Mathias W. Pletz, Maria J. G. T. Vehreschild, Silvia Würstle

**Affiliations:** ^1^Institute of Infectious Diseases and Infection Control, Jena University Hospital, Jena, Germany; ^2^German Cancer Consortium (DKTK), Partner Site Frankfurt, A Partnership Between DKFZ and University Hospital Frankfurt, Frankfurt, Germany; ^3^Goethe University Frankfurt, University Hospital Frankfurt, Department II of Internal Medicine, Infectious Diseases, Frankfurt, Germany; ^4^Department of Internal Medicine I, Endocrinology and Diabetology, University Hospital, Goethe University Frankfurt, Frankfurt, Germany; ^5^Leibniz Center for Photonics in Infection Research (LPI), Jena, Germany; ^6^Yale Center for Phage Biology and Therapy, Yale University, New Haven, CT, United States

**Keywords:** bacteriophages, phage therapy, diabetic foot ulcers, biofilm, 3D models

## Abstract

Diabetic foot ulcers (DFU) affect up to 15–25% of patients suffering from diabetes and are considered a global health concern. These ulcers may result in delayed wound healing and chronic infections, with the potential to lead to amputations. It has been estimated that 85% of diabetes-related amputations are preceded by a diagnosis of DFU. A critical factor in the persistence of this disease is the presence of polymicrobial biofilms, which generally include *Staphylococcus aureus*, *Pseudomonas aeruginosa*, and *Escherichia coli*. The involvement of diabetic comorbidities such as ischemia, hyperglycemia, and immune-compromised status creates a perfect niche for these bacteria to evade the body’s immune response and persist as biofilms. Bacteriophage therapy can target and lyse specific bacteria and is emerging as an effective treatment for biofilm-related infections. While this treatment shows promise in addressing chronic wounds, our current models, including animal and static systems, fail to capture the full complexity of DFU. Innovative approaches such as 3D bioengineered skin models, organoid models, and hydrogel-based systems are being developed to simulate DFU microenvironments more accurately in 3D without using *ex vivo* or animal tissues. These advanced models are critical for evaluating bacteriophage efficacy in biofilm-associated DFU, aiming to enhance preclinical assessments and improve therapeutic outcomes for DFU patients.

## Introduction

Diabetic foot ulcers, also known as DFU, are a severe global health concern which can affect up to 15–25% (Singh et al., 2005) of individuals suffering from diabetes. This condition can, in some cases, result in complications such as chronic infections, delayed healing of wounds and, in severe cases, lead to amputations (Edmonds et al., 2021). These comorbidities often restrict patient mobility and incur considerable costs (Tchero et al., 2018) A key factor in the perseverance and recurrence of DFU is the presence of bacterial infections in the form of sessile cells–biofilms (Afonso et al., 2021). Biofilms are structured microbial communities embedded in a self-produced extracellular matrix. This matrix protects the bacteria from antibiotics and immune responses, making infections associated with DFU difficult to eradicate (Costerton et al., 1999; Lewis, 2008). Conventional research methods often fall short in mimicking the complexity and resistance of biofilms present in actual human tissues. In the case of biofilms in patients suffering from DFU, the biofilm is not necessarily associated with one pathogen; it has been reported that these biofilms are polymicrobial in which the primary pathogens present are *Staphylococcus aureus*, *Pseudomonas aeruginosa*, and other Gram-negative pathogens, such as *Klebsiella* spp. and *Escherichia coli* (Sharifah Aisyah et al., 2019). These pathogens can take advantage of the comorbidities associated with diabetes, such as ischemia, immunosuppression, and a hyperglycemic microenvironment in the wound (Tatara et al., 2018; Zhang et al., 2022). This enables them to resist the host’s natural defenses and evade traditional antimicrobial therapies. Hence, novel therapeutic approaches must be elucidated (Bjarnsholt, 2013; Malone et al., 2017).

Bacteriophage therapy (BPT) is a treatment approach that was utilized in the early 20th century but took a back seat with the advent of antibiotics (Lin et al., 2017). BPT exploits lytic bacteriophages (phages), which can effectively infect and replicate within the targeted bacterial population before subsequently lysing the bacteria. Their simple nature renders them unable to propagate by themselves, relying instead on highly specific bacterial hosts for reproduction. This dependency fosters a dynamic co-evolutionary relationship between phages and their bacterial hosts, shaping the diversity and adaptation of both over time (Martinet et al., 2024). The use of phages to kill selected bacteria while sparing the beneficial microbiome of the human body has been proposed for the treatment of biofilm-associated infections (Cano et al., 2021; Chan et al., 2018; Doub et al., 2021; Doub et al., 2022; Ferry et al., 2021; Pirnay et al., 2024). It is well-known that phages encoding a depolymerase can effectively penetrate the biofilm by degrading the extracellular polymeric substance (EPS) using the depolymerase enzyme (Knecht et al., 2020). Several case studies have elucidated phages’ use treating chronic wound infections, including DFU (Kifelew et al., 2024; Morozova et al., 2024; Young et al., 2023). However, biofilm models including phages are needed to closely replicate the complexity of DFU. Commonly used methods rely on closed or open systems such as the static microtiter plates (closed system) and flow cells (open system) (Coffey and Anderson, 2014; Crusz et al., 2012; Henriksen et al., 2019; Sternberg and Tolker-Nielsen, 2006). These models offer high reproducibility and simplicity but do not reflect the complex features of DFU. Several animal models, such as porcine and murine models (Couturier et al., 2023; Seaton et al., 2015) can provide insight into the pharmacodynamics and phage-host interactions. However, their applicability is limited, as the complexity of DFU in humans cannot be fully replicated in animal models (Du et al., 2023). To better mimic the complexity of DFU, several new 3D methods are being developed for this specific microenvironment, including hyperglycemia, hypoxia, and polymicrobial infections. These new models offer a better simulation of the DFU microenvironment without the use of *ex-vivo* tissue models and animal models, challenging the previous consensus among researchers that wound healing is best studied using animal models (Pignet et al., 2024). Moreover, employing 3D models for biofilms can significantly reduce animal usage, aligning with the 3Rs principle: replacement, reduction, and refinement. New 3D models are, for example, 3D bioengineered skin models (Randall et al., 2018), organoid models (Hynds and Giangreco, 2013; Kretzschmar and Clevers, 2016), and hydrogel/alginate-based models (Sønderholm et al., 2017; Thaarup et al., 2023). These models are an emerging and promising way to incorporate phages into biofilm testing. This use of phages could ultimately enable the preclinical evaluation of phages for treating biofilm-associated DFU. Additionally, we advocate for the integration of omics-based approaches, such as metagenomic analyses of the DFU microbiome and proteomics (Liu et al., 2023; Schmidt et al., 2021), which, however, is not the focus of this review about 3D biofilm models.

## The use of 3D bioengineered skin as a model for DFU biofilms

Human skin is one of the most complex organs in the body, composed of many different cell types. Generally, the skin can be divided into three distinct layers: the hypodermis, dermis, and epidermis (Kolarsick et al., 2011). Human skin serves not only as a barrier shielding the body from environmental stimuli but also plays a crucial role in protecting against foreign invaders such as microbes due to the significant presence of the immune system on our skin (Bos et al., 1987). Biofilms have traditionally been studied in 2-dimensional setups such as microtiter plate biofilms, and new ways of representing the complexity of the skin need to be set. This is currently the case with 3D bioengineered skins, which can be used to model biofilms in a 3D setting. A multitude of synthetic skins can be developed, including wounded skin, atopic skin, skin cancer, and, interestingly, the fabrication of diabetic-skin models. In short, creating a 3D-engineered model involves multiple steps, combining biomaterials, cells, tissue engineering, and new manufacturing processes. To create synthetic skin, a scaffold is typically designed, using biocompatible materials such as collagen, hydrogels, and polymers. These materials provide structural support for cells and mimic the extracellular matrix of natural skin. Once the scaffold is prepared, various skin cell types, including fibroblasts and keratinocytes, are seeded onto it. 3D bioprinting offers a method to layer these cells in a manner that replicates the multilayered architecture of human skin. After the cells are positioned on the scaffold, the engineered skin is matured in specialized bioreactors that supply the necessary nutrients and environmental conditions to promote cell and tissue growth. This advanced technique enables the creation of complex skin models for research purposes (Ahn et al., 2023; Imran et al., 2024; Randall et al., 2018).

To the best of our knowledge, although never previously described in the literature for phages, these 3D bioengineered skin models offer, in addition to the general advantages of 3D models for biofilms, adaptability and customization for specific studies. These include varying scaffold compositions, skin types, the creation of polymicrobial biofilms, and the evaluation of different therapeutic approaches and application methods, with the added benefit of enabling quantification through bacterial burden measurement. This could ultimately be used for the study of phages which according to the studied literature has- never been explored. However, the use of synthetic skin as a model for biofilm studies is limited by several factors that must be carefully considered. First, the complexity of these models and the technical expertise required for their development demand highly skilled researchers with proficiency in tissue engineering and culture techniques. These requirements contribute to higher costs due to the need for specialized materials, advanced technologies, and time-intensive setups. Scaling up the production of this model for a more high-throughput screening remains a major challenge involving a significant cost effort and reducing the efficiency of large-scale studies. Additionally, challenges related to the longevity of bioengineered cells and the reproducibility of these models further underscore the limitations of synthetic skin as a research tool. Another point to consider is that while some models may incorporate immune cells, the complexity of the human immune response to biofilms in DFUs is not fully replicated. This simplification limits the accuracy of evaluating the immune-modulatory effects of phage therapy. Likewise, the absence of a vascular network in most bioengineered skin models limits the accurate representation of nutrients and oxygen supply to the biofilm, impacting biofilm development and treatment efficacy.

To summarize, 3D bioengineered skin models are a promising new way to visualize the complexity of DFU related biofilm infections. With the advent of BPT, this model can help researchers to understand how phages interact with the human cells and the biofilm formed. 3D bioengineered skin models, while offering advanced platforms to study biofilms and test therapeutic approaches such as phage therapy, present challenges like technical complexity and high costs.

## Organoids as a model to simulate biofilms in DFU

Organoids offer a promising approach for simulating biofilms in DFU. These advanced 3D models derived from stem cells can mimic a wide range of complex conditions, including but not limited to wound models, skin structures, and the intricate environments seen in cystic fibrosis, such as lung and intestinal systems (Lebeko et al., 2019; Lee and Koehler, 2021; Lombardi et al., 2024; Qu et al., 2021). Organoids more accurately represent the complex architecture and cellular structure of human tissues compared to traditional 2D cell cultures. The technique can bridge the gap between conventional biofilm models and the actual tissue infections in patients. By using the patient’s cells to generate organoids, researchers can recreate the microenvironment of DFU patients (Tong et al., 2024; Yang et al., 2018). This model enables scientists to observe the persistence, formation, and antimicrobial resistance of biofilms by closely mimicking the physiological conditions of a wound (Lehmann et al., 2019). Additionally, organoids enable the visualization of several DFU-related aspects, such as reduced blood flow and altered immune responses to bacteria, in a more human-relevant context.

Another method for visualizing organoids is the creation of an ulcer-on-a-chip. This technique utilizes microfluidics to replicate the skin environment, control fluid dynamics, and enable real-time monitoring of the process (Ejiugwo et al., 2021). This system can likewise be used to add bacteria to the microfluidic chamber to produce biofilms (Wei and Yang, 2023; Yuan et al., 2023).

Several studies have shown that phages can effectively be used with organoids (Lebeko et al., 2019; Yang et al., 2018), but to the best of our knowledge, studies relating to DFU biofilm are currently lacking. When integrated with organoid models, the interactions between phages and biofilm-forming bacteria can be studied in real-time, providing valuable insights into how phages may disrupt biofilm integrity and facilitate healing in DFU.

While organoids have the potential to provide a stable method for the modeling of 3D biofilms, there are still some limitations. Culturing these organoids is complex, as they are typically derived from stem cells or differentiated cells, which contributes to higher costs and additional challenges in their development. Additionally, while organoids can be used to analyze biofilms, visualizing biofilm formation within their 3D structure can be technically challenging. Imaging techniques such as confocal laser scanning microscopy (CLSM) may require specialized setups and can be hindered by the dense nature of both organoids and biofilms. Furthermore, several limitations related to the structural integrity of organoids must be considered, including their limited lifespan, variability between organoids, and the absence of complex host-pathogen interactions (Andrews and Kriegstein, 2022; Clevers, 2016). The scalability of organoid production currently limits their use in high-throughput screening assays. Ultimately, while organoids are derived from stem cells or patient samples, the ethical implications of their use should be carefully considered, particularly regarding informed consent and the potential for unintended consequences.

Despite these disadvantages, phages should be explored in combination with organoid models for DFU to understand better their potential in disrupting biofilms and promoting wound healing.

## Hydrogel models and alginate bead models

Hydrogel models have gained increasing importance in biofilm research over the past few years. Hydrogels are water-swollen networks of polymer chains that can mimic the extracellular matrix of tissues, offering a supportive environment for bacterial growth. The biocompatible materials of hydrogels allow for the entrapment of cells and bacteria. Collagen can be added to these hydrogels to facilitate cell adhesion, migration, and proliferation (Antoine et al., 2014). An incorporation of human cells into these models such as fibroblasts, fat cells, keratinocytes and immune cells further enriches this model (Fan et al., 2024). Customization of the model allows for the simulation of specific conditions, such as pH, oxygen tension, nutrient levels, and the presence of inflammatory cytokines (Kurnia et al., 2012; Mavris and Hansen, 2021). This versatility makes the model ideal for testing various therapeutic approaches, including phages.

Alginate, a biopolymer derived from seaweed, is particularly advantageous for creating these models due to its biocompatibility and ability to form hydrogels. When combined with bacterial strains isolated from DFU patients, alginate promotes biofilm formation, enabling researchers to study the structural and functional aspects of biofilms in a controlled environment. This setup allows for the observation of biofilm development, maturation, and resistance mechanisms against antibiotic treatments (Dsouza et al., 2022; Kandemir et al., 2018; Pabst et al., 2016; Pham et al., 2023; Stewart et al., 2015; Zheng et al., 2021).

The alginate bead model is a method used to model bacterial biofilms encapsulated within alginate, which could potentially be used to study DFU-related biofilms with phages. In this approach, an alginate solution is mixed with a bacterial culture, and the addition of calcium ions causes the alginate to cross-link, forming beads that encapsulate the bacteria. This method offers several advantages, including the biocompatibility of alginate with the bacteria, high throughput capabilities, and the ability to visualize simulated biofilm formation through imaging (Ali et al., 2024; Sønderholm et al., 2017). In most of the studied cases the use of alginate beads has been used as a common mean of delivery of phages (Abdelsattar et al., 2019; Chen et al., 2023; Gomez-Garcia et al., 2021).

Generally, the use of alginate and hydrogels is related to the delivery of antibiotics or phages, and several studies have shown that this method is reliable and can easily be used in a laboratory setting (Barros et al., 2020; Dumville et al., 2013; Gomez-Garcia et al., 2021; Ko and Liao, 2023).

Regarding biofilm modeling with alginate and hydrogels, the main disadvantages include variability in the models, which can vary significantly depending on the preparation method and the specific material used, and the complexity of biofilm dynamics, which may not be fully captured or accurately represented in these systems. This could be due to the complexity of quantifying the biofilms embedded into these models, and measuring biofilm biomass and structure in 3D hydrogel/alginate models can be challenging and requires sophisticated techniques like confocal microscopy and image analysis. Hydrogel and alginate models generally lack the complexity of cellular interactions found in the DFU microenvironment. The absence of immune cells, fibroblasts, and other cell types limits the accuracy of evaluating the host response to infection and treatment.

**FIGURE 1:**
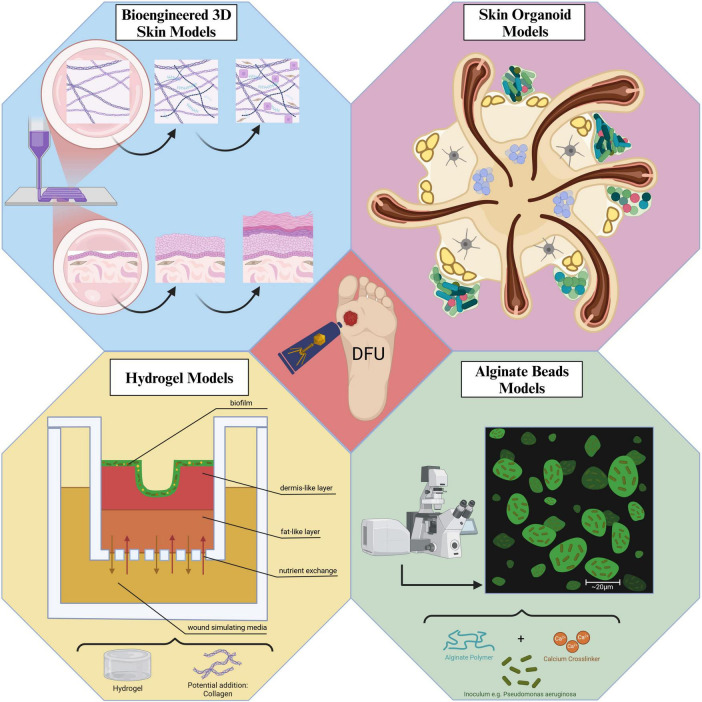
Graphical summary of the different ways of modeling biofilms in DFU associated infections.

In the context of phage delivery, the physical barrier created by biofilms requires thorough investigation to determine its impact on the penetration of beads or hydrogels containing the phages into the wound or biofilm. A general summary and overview of the models is depicted in Figure 1.

## Conclusion and future perspectives

In conclusion, the modeling of biofilms in DFU has significantly advanced over time, from 2D models to 3D. These new types of modeling have given us valuable insight into the complexity of wound healing and the challenges in these chronic wound infections. Biofilms are well known to enhance bacterial resistance toward antimicrobial treatments; thus, understanding the dynamics of DFU is important for developing future therapeutic strategies. Current models, such as three-dimensional skin engineering, organoids, and hydrogel/alginate systems, have allowed researchers to mimic the human environment better, facilitating the study of biofilm interactions, treatment efficacy, and overall wound healing process. While here we covered the published literature of 3D biofilm models capable of modeling phage therapy in DFU while expanding on the limitations, several new areas could be explored in the future. To overcome the limitations of 3D models, advancements in bioprinting technology are needed to enhance overall precision, enable higher-throughput screening, and ultimately reduce costs. Standardizing 3D models through well-defined protocols is essential to ensure reproducibility and comparability. Additionally, the exploration of novel biomaterials and hydrogel formulations can contribute to more realistic and versatile models. Lastly, integrating advanced imaging techniques for more accurate visualization of biofilm structures, along with sophisticated computational models to predict biofilm behavior, will further enhance the reliability and applicability of these systems.

Looking ahead, the use of phages in biofilm modeling for DFU offers the potential for novel treatment strategies. These highly specific viruses can disrupt biofilm integrity and enhance the effectiveness of traditional antimicrobial therapies. Ultimately, phage-based approaches could improve our understanding of the complexity of DFU wound modeling and help explore their synergistic effects on biofilm clearance and wound healing. Additionally, the continued use of 3D bioengineered skin models and organoids, along with patient-specific samples, microbiomes, and biofilm compositions, will enable more personalized treatment approaches. The continued use of 3D bioengineered skin models, organoids, and hydrogel/alginates can further enhance the flexibility and functionality of tailored DFU treatment regimens.

A summary of current clinical trials related to DFU, and phages documented in *clinicaltrials.gov* (Table 1) reveals that relatively few trials have been conducted, with several still ongoing and recruiting participants. Notably, only two phase I trial have been completed thus far, targeting ulcers with multi-species phage cocktails, both not showing adverse events associated to the treatment (Nir-Paz et al., 2022; Rhoads et al., 2009). Although clinical trials are limited, several case reports about phage therapy against DFU infections are published (Kifelew et al., 2024; Young et al., 2023). Notably, in a case series from the UK with ten patients treated with phages, an experienced clinical team observed that nine out of ten patients seemed to benefit from adjunctive phage therapy, with no adverse effects reported. In six patients, phage therapy aided in resolving infections and saving limbs. A seventh patient’s soft tissue infection resolved, but osteomyelitis required amputation. Eight patients saw eradication of *Staphylococcus aureus* in a polymicrobial infection, and a ninth showed improvement before phage therapy stopped early due to an unrelated event. One patient with a weakly susceptible *Staphylococcus aureus* isolate did not show a significant response (Young et al., 2023). In general, the use of phages as a compassionate mean to treat difficult to treat infections can be seen in a multitude of cases (Fish et al., 2018; Onallah et al., 2023; Pirnay et al., 2024).

**TABLE 1:** Overview of completed, ongoing, or withdrawn clinical trials involving DFU and phages.

Target bacteria	Study start/estimated or actual completion	Treatment	Outcome	Number of enrolled patients/estimated number	References
*Pseudomonas aeruginosa*, *Escherichia coli*, and *Staphylococcus aureus*	09-2006/05-2008	Phase I randomized double-blind controlled trial using “WPP-201” for the treatment of venous leg ulcers. No focus on diabetes patients.	Completed. No adverse events linked to the study product, and no significant differences in adverse event frequency, healing rate, or healing frequency between the test and control groups.	64	Available at: https://clinicaltrials.gov/NCT00663091
*Pseudomonas aeruginosa*, *Staphylococcus aureus*, *Acinetobacter baumannii*	03-2021/09-2022	Phase I/IIa randomized double-blind trial using “TP-102” in diabetic foot ulcers	Completed. No severe adverse events associated with the treatment were observed.	20	Available at: https://clinicaltrials.gov/NCT04803708
*Staphylococcus aureus*	06-2022/estimated completion 08-2024	Phase I/II randomized double-blind controlled trial for diabetic foot ulcers infected by *Staphylococcus aureus*	Status unknown	60 (estimated)	Available at: https://clinicaltrials.gov/NCT02664740
*Staphylococcus aureus*	03-2021/estimated completion 09-2022	Phase I/II randomized double-blind controlled trial for the management of infected foot ulcers in diabetes	Withdrawn	Not known	Available at: https://clinicaltrials.gov/NCT04289948
*Staphylococcus aureus*	11-2021/Estimated completion 12-2024	Phase IIb randomized double-blind controlled trial for diabetic foot osteomyelitis	Recruiting	126 (estimated)	Available at: https://clinicaltrials.gov/NCT05177107
*Staphylococcus aureus*, *Pseudomonas aeruginosa*, *Acinetobacter baumannii*	11-2023/estimated completion 12-2024	Phase IIb randomized double-blind controlled trial using “TP-102” in patients with diabetic foot infection	Recruiting	80 (estimated)	Available at: https://clinicaltrials.gov/NCT05948592

In summary, the continued evolution of biofilm models coupled with phage therapies and new advancing engineering techniques gives us significant promise in improving the outcome of DFU management and addressing the persistence of biofilm-associated infections. Research into addressing these issues will be pivotal for elucidating these complex interactions and pave the way for novel therapeutic approaches to improve the healing process in DFU patients.
